# Supporting Primary Care Professionals to Stay in Work During the COVID-19 Pandemic: Views on Personal Risk and Access to Testing During the First Wave of Pandemic in Europe

**DOI:** 10.3389/fmed.2021.726319

**Published:** 2021-09-10

**Authors:** Marta Wanat, Melanie Hoste, Nina Gobat, Marilena Anastasaki, Femke Böhmer, Slawomir Chlabicz, Annelies Colliers, Karen Farrell, Maria-Nefeli Karkana, John Kinsman, Christos Lionis, Ludmila Marcinowicz, Katrin Reinhardt, Ingmarie Skoglund, Pär-Daniel Sundvall, Akke Vellinga, Herman Goossens, Christopher C. Butler, Alike van der Velden, Sibyl Anthierens, Sarah Tonkin-Crine

**Affiliations:** ^1^Nuffield Department of Primary Care Health Sciences, University of Oxford, Oxford, United Kingdom; ^2^Department of Family Medicine and Population Health, University of Antwerp, Antwerp, Belgium; ^3^Laboratory of Medical Microbiology, Vaccine & Infectious Disease Institute, University of Antwerp, Antwerp, Belgium; ^4^Clinic of Social and Family Medicine, Faculty of Medicine, University of Crete, Crete, Greece; ^5^Institute of General Practice, Rostock University Medical Centre, Rostock, Germany; ^6^Department of Family Medicine, Medical University of Bialystok, Bialystok, Poland; ^7^School of Medicine, National University of Ireland, Galway, Ireland; ^8^European Centre for Disease Prevention and Control (ECDC), Solna, Sweden; ^9^Department of Obstetrics, Gynaecology and Maternity Care, Medical University of Bialystok, Bialystok, Poland; ^10^General Practice/Family Medicine, School of Public Health and Community Medicine, Institute of Medicine, Sahlgrenska Academy, University of Gothenburg, Gothenburg, Sweden; ^11^Research, Education, Development & Innovation, Primary Health Care, Gothenburg, Sweden; ^12^Health Research Board (HRB) Primary Care Clinical Trials Network, Galway, Ireland; ^13^Laboratory of Clinical Microbiology, Antwerp University Hospital, Edegem, Belgium; ^14^National Institute for Health Research (NIHR) Health Protection Research Unit in Healthcare Associated Infections and Antimicrobial Resistance, University of Oxford in Partnership With Public Health England, Oxford, United Kingdom; ^15^Julius Center for Health Sciences and Primary Care, University Medical Center Utrecht, Utrecht, Netherlands

**Keywords:** primary care/general practice, setting of care, qualitative analysis, remote, patient-centred care, healthcare profession

## Abstract

**Background:** Minimising primary care professionals' (PCPs) risk of SARS-CoV-2 infection is crucial to ensure their safety as well as functioning health care system. PCPs' perspectives on the support they needed in the early stages of a public health crisis can inform future preparedness.

**Aim:** To understand PCPs' experiences of providing care during the COVID-19 pandemic, with focus on personal risk from COVID-19 and testing.

**Design and Setting:** Qualitative study using semi-structured interviews with PCPs in England, Belgium, the Netherlands, Ireland, Germany, Poland, Greece and Sweden, between April and July 2020.

**Method:** Interviews were analysed using a combination of inductive and deductive thematic analysis techniques.

**Results:** Eighty interviews were conducted, showing that PCPs tried to make sense of their risk of both contracting and severity of COVID-19 by assessing individual risk factors and perceived effectiveness of Personal Protective Equipment (PPE). They had limited access to PPE yet continued providing care as their “duty.” Some PCPs felt that they were put in high-risk situations when patients or colleagues were not flagging symptoms of COVID-19. Not having access to testing in the initial stages of the pandemic was somewhat accepted but when available, was valued.

**Conclusion:** Access to adequate PPE and testing, as well as training for staff and education for patients about the importance of ensuring staff safety is crucial. Given PCPs' varied response in how they appraised personal risk and their tolerance for working, PCPs may benefit from the autonomy in deciding how they want to work during health emergencies.

## Introduction

Healthcare professionals (HCPs) are at high risk of contracting COVID-19 (1) and data from Europe shows that as many as 20% of HCPs might have been infected (2). The occurrence of “long COVID” also appears higher among HCPs, possibly because of their increased exposure to SARS-CoV-2 (3). There is incomplete data on deaths related to COVID-19 among HCPs (4, 5) but estimates from July 2020 show that at least 3,000 HCPs in 79 countries had died by then because of COVID-19 (5) and more recent data show that over 600 HCPs, including PCPs, died in the UK alone (6).

Preventing transmission of SARS-CoV-2 in healthcare workplaces involves a number of strategies including infection prevention protocols, that include use of Personal Protective Equipment (PPE), and testing for infection along with isolation for people who test positive (7). In the initial stage of the pandemic, a primary focus for prevention of COVID-19 was on hospital workforce, which meant that, in this time of shortages, primary care had insufficient access to PPE (8, 9).

In a number of countries, PCPs also experienced limited access to testing (10), which had consequences for staffing levels. A recent study in the UK showed that sickness absence rates among HCPs in April 2020 rose above the 10-year average (11), suggesting that not only staff with confirmed COVID-19 symptoms but also staff with potential or suspected infections were self-isolating, having potentially severe consequences, at a time when healthcare systems had already been under severe pressure (10).

The focus on personal risk and its impact in secondary, rather than primary care, has been reflected in research published on this topic. A recent qualitative study with HCPs working in hospital settings found initially limited training on using PPE, anxiety brought by shortages and confusion from changing guidance related to PPE (12). There is limited research on PCPs' views of working during the pandemic in relation to managing personal risk. A recent UK survey highlighted that HCPs working in primary and community care settings were dissatisfied with their supply of PPE (13) and had unresolved concerns in relation to required PPE (13). There is even more limited insight into HCPs' views on COVID-19 testing, in any setting in relation to perceived need, availability, and perceived value.

Minimising the risk of infection among healthcare workforces is crucial to protect the physical and mental health of healthcare professionals and to prevent healthcare systems from becoming overwhelmed (14). Our study addressed the current gap by exploring the PCPs' views and experiences of providing care during the COVID-19 pandemic (15). We aimed to understand their views on perceived personal risk from COVID-19 and on COVID-testing in order to identify lessons facilitating safety of PCPs and functioning health care systems in preparedness for potential future health emergencies.

## Methods

### Design, Setting, and Recruitment

This was a qualitative study based on semi-structured interviews carried out with PCPs delivering care during the first wave of the COVID-19 pandemic. We used qualitative methods because we aimed to understand experiences and important issues from the perspective of PCPs. In conducting the study, we drew on the Platform for European Preparedness Against (Re-)emerging Epidemics (PREPARE) primary care research network. Longstanding relationships with network coordinators enabled the rapid set up and delivery of this research at scale.

PCPs were recruited from eight European countries, which included England, Belgium, Ireland, the Netherlands, Germany, Poland, Sweden, and Greece. Participating countries were selected as they varied in number of confirmed cases of COVID-19 in March 2020, organisation of health systems and geographical locations in Europe. Each country had a network coordinator who had access to primary care sites, from which local PCPs were recruited. Interviewers in each country invited PCPs to the study by email or telephone. More details on selection of countries is presented elsewhere (15).

### Data Collection

Prior to the start of data collection, researchers completed a live, online study training to ensure consistency in interview approach. This included training in the aims of the study and key topics of the semi-structured topic guide. Interviewers led data collection in their country and collected data in local languages. When participating PCPs had given consent to take part, the interviews were conducted over the telephone or face-to-face, audio recorded, transcribed verbatim and those undertaken in countries other than the UK and Ireland were translated into English.

### Data Analysis

M.W. analysed all data using a combination of deductive and inductive thematic analysis (13). Analysis started with data from England, Belgium, and the Netherlands, as these interviews were conducted first. The first stage involved a deductive analysis; transcripts from the three aforementioned countries were read line-by-line and were coded into an *a priori* framework of 14 categories was based on the topic guide. We then proceeded with an inductive analysis; we coded data within each of the 14 categories line by line in order to create sub-categories, and then grouped these to form themes and sub-themes. We used this thematic framework to analyse data from the other countries. We used a constant comparative approach to analysis whereby we as researchers moved back and forth between the data and emerging themes until all data had been analysed (16). Any data which did not fit into the initial themes were discussed within the research team and themes were edited, changed, added and renamed on a regular basis to ensure that they represented data across all countries. To further ensure rigour, the ongoing analysis was discussed within the multidisciplinary study team and all interviewers in each country on a monthly basis to ensure understanding of the local context, where relevant to interpreting findings. NVivo 12 was used to facilitate data analysis (17). This article adheres to the Consolidated Criteria for Reporting Qualitative Research (COREQ) guideline.

## Results

We conducted 80 interviews between 2nd April and 2nd July 2020; these lasted between 17 and 86 min (mean 35 min). Participant characteristics are summarised in Table 1.

**Table 1:** Characteristics of participants by country.

	**England**	**Belgium**	**Netherlands**	**Ireland**	**Germany**	**Greece**	**Poland**	**Sweden**	**All countries**
	**(*N* = 11)**	**(*N* = 10)**	**(*N* = 10)**	**(*N* = 10)**	**(*N* = 9)**	**(*N* = 10)**	**(*N* = 10)**	**(*N* = 10)**	**(*N* = 80)**
Age range (mean)	29–62 (47.3)	29–63 (44)	33–56 (45.8)	32–60 (43.3)	29–61 (43.2)	26–51 (39.8)	29–59 (49.2)	31–58 (43.5)	26–63 (44.5)
Female sex % (*N*=)	72% (8)	50% (5)	60% (6)	60% (6)	56% (5)	80% (8)	90% (9)	70% (7)	68% (54)
GPs	7/11	10/10	10/10	10/10	4/9	3/10	8/10	5/10	57/80
Nurses	3/11	N/A	N/A	N/A	1/9	4/10	2/10	4/10	14/80
Other HCPs	N/A	N/A	N/A	N/A	2 GP trainees; 1 physician assistant; 1 paediatrician working in primary care	1 assistant nurse, 1 social worker, 1 paediatrician working in primary care	N/A	1 Nurse assistant (responsible for testing patients for COVID-19)	8/80
Years of experience	1–32 years	5–38 years	2.5–19 years	4–33 years	6–37 years	3–20 years	4–37 years	9–31 years	1–38 years
Tested for COVID-19 at time of interview	None	3/10	2/10	6/10	3/9	None	1/10	3/10	18/80
Tested positive	N/A	3	None	1	None	N/A	None	None	4

We identified four themes. These were:

1. PCPs' sense of personal risk,
2. PCPs' views of COVID-19 testing,
3. Transformation of primary care delivery and PCPs' experiences of these changes,
4. Navigating a new relationship with patients.

Given the depth of data gathered across countries, here we report data on themes 1 and 2, which provide an insight into the barriers and facilitators to PCPs staying in work during the pandemic. The remaining themes have been reported elsewhere (15, 18). Key information regarding the timing of the interviews and contextual information for each country is presented in Table 2.

**Table 2:** Timing of interviews and contextual information by country.

	**England**	**Belgium**	**Ireland**	**Netherlands**	**Germany**	**Greece**	**Poland**	**Sweden**
Dates of interviews	2nd April−13th May	6th−30th April	10th−29th April	1st−22nd May	1st May−2nd July	18th May−1st June	30th May−19th June	5th−17th June
Dates of strict lockdown	23rd March−10th May	13th March−4th May	12th March−18th May	9th March−11th May	16th March−20th April	16th March−4th May	12th March−3rd May	N/A
Timing of interviews	During lockdown	During lockdown	During lockdown	During lockdown (+1 week)	1–10 weeks after strict lockdown lifted	2–4 weeks after strict lockdown lifted	2–8 weeks after strict lockdown lifted	N/A
Number of cases in each country at the time of the interviews	88 625 (14th April 2020) (19)	30 589 (14th April 2020) (19)	10 647 (14th April 2020) (19)	26 551 (14th April 2020) (19)	169 218 (10th May) (20)	2 710 (10th May) (20)	31 620 (21st June) (21)	58, 878 (21st June) (22)
Number of infected people per 100, 000 in each country around the time of interviews (14/7)	110.7 (23rd April 2020) (23)	163.8 per 100 000 (23rd April 2020) (23)	210.7 (23rd April 2020) (23)	8.3 (30th June 2020) (24)	218 (31st May) (25)	29 (10th June) (26)	11.5 (30th June) (24)	149.4 (30th June) (24)

### Theme 1: PCPs' Sense of Personal Risk

#### Sub-Theme 1: Implementation of Protective Measures and Beliefs About Their Effectiveness

PCPs across all countries described implementing various infection control procedures in GP practises in order to minimise the risk of infection for themselves and for patients. They reported extensive efforts to secure PPE supplies for their staff, and experienced inadequate supplies. This resulted in clinicians seeing patients without adequate PPE or reusing equipment. PCPs across all countries felt that lack of PPE was putting them at risk and for some, this had negative impact on their mental health.

Views about perceived effectiveness of PPE seemed to be linked to how care was organised in different settings. PCPs in Belgium, and the Netherlands felt that because of the perceived sufficient availability of PPE and strict safety protocols, COVID hubs (where patients suspected of COVID-19 were seen) were overall a safer place to work in than primary care practises (where there was usually shortage of PPE). In contrast, some PCPs in the UK, Ireland, and Sweden highlighted that they feared working in the COVID hubs and tents (Sweden) as they were most likely to be in contact with patients most likely to have COVID-19, thus indicating that access to PPE was not perceived as sufficient to reassure them.

*When the container] was very narrow, you're very close to the patient and the patient coughs, then it's not particularly funny to be standing there; you just wanted to get finished and get out of there. [P1, GP, Sweden]*

#### *Sub-Theme 2: Appraising Individual Risk Factors for Contracting* SARS-CoV-2 *and the Consequences of Getting Infected*

PCPs tried to appraise their risk of both getting infected and its potential consequences based on a number of factors such as their age, sex, overall health, or ethnicity. For example, seeing oneself as young, healthy and without underlying health issues, made PCPs felt reassured. In contrast, being older, with underlying health conditions or from an ethnic minority community was worrying. Of note is that PCPs differed in their confidence in whether they would get seriously ill from COVID-19. While some felt almost “invincible,” others expressed uncertainty around their risk and their appraisal of risk seemed to go “back and forth.”

*God only knows, like I don't know, I would hope that I would just have a mild. […] I'm in my middle 40s so I'm not in an at risk group as such, but you know unfortunately there have been people, I'm certainly not the youngest candidate either you know, so yeah look I would hope I would just have a mild illness [P4, GP, Ireland]* *Actually, I've never really been afraid of [getting COVID] because I don't have any risk factors myself. [P8, GP, Belgium]*

Secondly, PCPs also took into account “local evidence” related to the impact of the COVID-19 pandemic. Some PCPs viewed the response to the pandemic as overcautious, especially if there was low local incidence. In contrast, hearing of deaths of other PCPs or storeys about severe situations in other countries increased their concerns.

Thirdly, the very small number of PCPs who had had confirmed COVID-19, described how their views related to possible consequences of getting infected were somewhat challenged as a result of being infected.

#### Sub-Theme 3: Negotiating the “Need” to Fulfil One's Duty

PCPs often described their sense of personal risk in relation to their sense of duty to work during the pandemic. They spoke of their feelings of guilt if not “stepping up” and a sense of satisfaction when being able to contribute to fighting the pandemic.

*I thought-okay, it sounds a bit Utopian-that once in our lives, the earth asked us, after all, and the whole world, to cope with a pandemic! Shall we not answer to this?[…], I was really happy that we can contribute! Not that I was not afraid! Dear God, these have nothing to do with each other [P2, Greece]*

There were also notable differences in responses. Some PCPs felt that risk is an inherent part of their job, or saw their risk as relatively low and hence seemed to accept changes to their roles.

*As a doctor I don't really have an issue with it, that's the nature of the job, that's the nature of what I was trained to do. […] There's always a risk. [P5, GP, Ireland]*

Others felt that the risk had increased significantly and felt that they were being asked to give more than they were willing. For example, some PCPs in England and Ireland described their gratitude toward colleagues who volunteered to work in the hubs (which they considered as high-risk settings) and some expressed their worries of being called to work there. Others almost felt resigned to accepting the need to work in situations which they perceived risky, to protect other colleagues, while bearing the burden of potential consequences for their own and their family's safety.

*I think it's our job unfortunately. I'm not delighted about the idea of working in COVID hubs to be honest with you., […] obviously if something happens to them [kids], you know it was a really stupid move on your behalf to go and work there and yet when you kind of know the odds are lower for you than they would be for your 50/60 year old colleague you kind of have to go with it I think. [P3, Ireland]*

Some PCPs in Poland, Greece and Sweden felt that their sense of duty was at times being taken advantage of, and felt that they were not looked after or their safety ignored, for example, when patients were concealing their symptoms in triage.

*In the beginning it was a sense of responsibility. Then came the fear that we might catch something and the frustration when some people came here without warning and without saying that they had a fever [P1, GP, Greece]*

Notably, some PCPs also reflected on the more implicit pressure to contribute to the pandemic and work in situations perceived as risky, to support their colleagues. It seems that at times collective responsibility also meant collective pressure to work together.

#### Sub-Theme 4: Tensions in Understanding and Managing Risk as Individual or Collective Responsibility

PCPs felt that their colleagues largely adhered to infection control procedures and there was a sense of collective responsibility and peer support to ensure safety of all staff, including non-clinical colleagues. Some PCPs who fell ill described how they felt supported by their colleagues when they took on their duties in their absence and checked on them. In addition, some PCPs who felt that they were at higher risk of COVID-19, for example due to being pregnant or belonging to certain ethnic groups, described the process of negotiating reduced patient contact with their colleagues and management. At the time, the evidence regarding risk within this groups was unclear.

PCPs in Poland and Belgium described instances of lacking shared understanding of the risk involved where some colleagues did not always follow appropriate protocols, for example, not wearing facemasks or inviting patients with respiratory tract infection (RTI) symptoms into the clinic. They found it upsetting as they felt it put everyone at risk.

*The doctor who invited such a patient didn't even inform the employees who were letting him in about the risk […] Such situations were very stressful. The manager intervened but he was repeatedly ignored. [P9, GP, Poland]*

PCPs in Belgium, the Netherlands and Germany also highlighted that some patients, or their colleagues triaging patients, who at times included administrative staff, unknowingly ignored risk symptoms and thereby putting PCPs at higher risk.

### Theme 2: Views of Testing

#### Sub-Theme 1: Perceived Need for Testing

At the time of the interviews, in Ireland, Sweden, Germany, Belgium, the Netherlands, and Poland it was possible for healthcare professionals who had symptoms and/or were exposed to a patient with COVID-19 to access testing. PCPs had varied views in relation to the need for testing which were not always explained by just country-specific guidelines but also other factors. Firstly, the majority of PCPs felt that rapid access to testing was important when displaying symptoms. PCPs in countries who had access to testing and had experienced symptoms, were grateful for this opportunity as it allowed them to continue working. PCPs in Ireland highlighted however, that access to testing for PCPs was limited in comparison to colleagues in secondary care or they had to wait a long time for results, which meant that they had to remain working from home, which in turn had impact on their colleagues' workload. PCPs in the Netherlands also described constantly changing regulations related to who was eligible for testing. In contrast, some PCPs in England and Greece who had symptoms but did not have access to tests felt that it had an emotional impact on them as they wanted to know whether they contracted the virus.

*There was a lot of unhappiness about not being tested. I felt a mixture of things. First of all, you'd like to know. We had trivial symptoms after my son [developed symptoms] […] they were so minor I don't know if they meant anything, but we weren't tested, and I think, emotionally, what does that mean? [P1, GP, England]*

Others tried to rationalise the lack of testing for primary care, highlighting that colleagues from secondary care should be prioritised as they were more at risk.

Secondly, views on the need for testing seemed to be linked to the perceived risk of getting COVID-19. Those who perceived their risk as low seemed to accept the lack of or limited access to testing. In contrast, PCPs who felt at higher risk of getting infected felt disappointed that they did not have access to testing.

*All of us should be tested, no matter if we have symptoms or not and if we can prove being in contact with an infected person or not. This is most important. [P1, Poland, Nurse]*

#### Sub-Theme 2: Doubts and Uncertainties Surrounding Testing

Early experiences of accessing testing also reflected emerging knowledge on the nature of the virus, its symptoms and transmission routes. For example, some PCPs in Ireland who decided to get tested, highlighted their hesitancy to get tested initially because of not displaying typical symptoms. Some PCPs also expressed uncertainty when one should be tested and found guidelines confusing and some sought clarification and advice from colleagues, which at times led to not getting tested.

Some PCPs also expressed their doubts about whether testing was reliable, both for symptomatic and asymptomatic individuals and some felt that getting tested was not necessarily worth it if done as a one-off rather than on regular basis.

*At the start you assumed, well that'll come, that we're all tested. But in the end, it also, yeah, quickly became clear, yeah, today you test negative, the next day you can be infected. (I: Mh.) What's the point? [P5, GP, Germany]*

## Discussion

### Summary

To our knowledge, this is the first qualitative study exploring PCPs' views of personal risk from COVID-19 and testing during the first wave of the pandemic. We found that PCPs reported suboptimal availability of PPE, which, for some, had a negative impact on their mental health. PCPs tried to make sense of their risk by assessing their individual risk factors for developing serious illness from SARS-CoV-2 infection and perceived effectiveness of PPE. Despite their worries, they often felt that it was their duty to continue providing care. Not having access to testing in the initial stage of the pandemic was somewhat understood but caused anxiety and when available, was mostly valued.

### Strengths and Limitations

This study into PCPs' perceptions of personal risk and testing, highlights key lessons for ensuring that PCPs remain safe during health emergencies. Additionally, this study is unique as it sampled PCPs from different countries with varied health contexts, highlighting how external factors such as organisation of primary care delivery, also influenced PCPs' sense of personal risk. Despite a large number of interviews overall, the number of interviews with PCPs who had been infected was small. Future studies could focus on PCPs with these experiences to gain further insights into these issues to identify issues which could be relevant in future health emergencies. Interviews with PCPs over time would also be useful to examine how their sense of personal risk and needs related to COVID-19 might have changed.

### Comparison With Existing Literature

In line with other reports showing that HCPs including PCPs had insufficient access at this early stage in the pandemic to PPE (5, 8, 9), we found that PCPs reported having to work without adequate PPE, which they felt put them at higher risk for getting infected and at times had a negative impact on their mental health.

Our study also highlighted that PCPs, despite personal risk, often talked about their sense of duty to continue delivering care. This seemed to be linked to how they saw their role as a healthcare professional. Recent studies described that some HCPs felt that they were expected to work in the face of unknown risk of infection, while not realising the potential long-term consequences of getting infected (27–30). In our study, PCPs seemed to accept working during the pandemic if they perceived the risk to be low, felt that some risk was part of their job, or felt satisfaction from contributing to helping fight the pandemic. In contrast, some felt that they were being asked to work in situations or settings in which they were not comfortable with, that they were put at risk by others and, at times, felt they had “no choice” but to continue working. Previous studies highlighted the importance of autonomy of healthcare professionals in relation to various aspects of providing care in the context of COVID-19. A study in the UK found that over 80% of surveyed PCPs felt that they were given sufficient autonomy to tailor their services to fulfil the needs of their patients and staff, which they valued (31) while also expressing desire for autonomy in the future. Another study highlighted differences in healthcare professionals' attitudes toward COVID-19 vaccines, showing that while some were keen to advocate vaccination to patients, others felt that it was not their role and decision should be left to the patient (32). However, the issues related to the importance of giving PCPs autonomy related to their personal safety at work during the pandemic have not been previously reported. Our study showed that PCPs were making decisions about working during the pandemic and the detrimental impact it had on them as well, as they felt that at times, they had limited autonomy related to that. The consequences of working in conditions which put one's health at risk, and the continuous threat to one's health and life are increasingly recognised, including the particular risk of suffering moral injury (33), and the impact this can have on the quality of care delivered (34).

Our study also found that PCPs who had access to testing valued this as they could get reassurance and continue working. In contrast, not having access to testing when having symptoms was difficult for PCPs. Another study highlighted anticipated benefits of providing testing such as preventing unnecessary isolation and staff not feeling obliged to continue working despite having symptoms (35), while a Danish study examined views of healthcare professionals while waiting for COVID-19 test results, and found that PCPs felt at times guilty about requesting a test as that had an impact on the workload of other colleagues, while feeling it was important to be cautious and protect their colleagues and patients from getting infected (36). Our study also highlights additional negative impact of lack of testing on PCPs, including impact on others' workload and not being able to know whether they were infected or not. It also highlighted the impact of emerging evidence in relation to testing, as some PCPs described doubts or uncertainties about the value of testing. Finally, even in the early stage of the pandemic, some PCPs wanted to have regular access to testing.

### Implications for Research and Practise

PCPs had limited access to PPE yet continued providing care. Some PCPs felt that they were put in high-risk situations when patients or colleagues were not reporting or flagging symptoms of COVID-19. The access to PPE is essential but in order to ensure staff safety, there is the need for information and education of the whole workforce to be vigilant about risk. Consistent messages from management highlighting the value of staff and consequently patients' safety might also be important, alongside education for patients and health messages, highlighting the need to protect the workforce and collective responsibility (37).

Lack of PPE, and, being asked or feeling compelled to work in conditions which PCPs felt uncomfortable with, had a negative impact on some PCPs. For those who accepted it, it is worth considering whether they would have done so, if they knew how long they would have to work under difficult circumstances. The issues around PCPs sense of duty and what is expected of them have not been discussed in detail previously. The usefulness of positioning PCPs as “heroes” has been challenged (38). Some highlighted that while the hero narrative was initially intended to reflect the appreciation toward healthcare workers, it may have been in fact detrimental as it often meant that PCPs were implicitly expected to accept unnecessary risks (39), and accept working in demanding conditions over a significant amount of time. Instead, mental health of staff needs to be a priority (38–40) which may mean allowing HCPs to take time off to prevent staff from taking sick leave or even leaving the healthcare service (41).

Finally, access to testing is also an essential step in ensuring safety of PCPs and the need for low-threshold access to testing alongside decisions about removal or return to work are crucial (7). Again, the lack of testing for the primary care workforce further reflects the initial lack of attention paid to this group of HCPs, which needs to be addressed when preparing healthcare systems for future health emergencies.

## Conclusions

PCPs tried to make sense of their risk by assessing a number of factors for both getting infected and developing serious illness from COVID-19. As a result, there was a great variation among PCPs in relation to how concerned they were to work during the pandemic, suggesting that PCPs have different role identities. Regardless of how worried they were, they often felt compelled to work and that it was their duty to continue providing care. Not having access to testing in the initial stage of the pandemic was somewhat understood and when available, was valued. Our study highlights that access to adequate PPE and testing, as well as training for staff and education for patients is essential part of preparedness for future pandemics. Given PCPs varied response to how they viewed their personal risk and their tolerance for working during the pandemic, PCPs should also be given an autonomy in being able to have a say in how they want to work during health emergencies.

## Data Availability Statement

The datasets for this article are not publicly available because the data are transcripts of interviews that reflect the views of individuals and complete anonymization cannot be guaranteed.

## Ethics Statement

The study was reviewed and approved by South Central-Berkshire Research Ethics Committee (ref. 20/SC/0175). The patients/participants provided their written informed consent to participate in this study.

## Author Contributions

MW and MH was responsible for acquisition, analysis and interpretation of data, and as well as drafting of the manuscript. NG, FB, SC, AC, KF, M-NK, JK, CL, LM, KR, IS, P-DS, AVell, HG, CB, and AVeld was responsible for acquisition, analysis and interpretation of data, as well as reading, and critically reviewing the manuscript. SA and ST-C was responsible for concept and design, acquisition, analysis and interpretation of data, as well as reading, and critically reviewing the manuscript.

## Conflict of Interest

The authors declare that the research was conducted in the absence of any commercial or financial relationships that could be construed as a potential conflict of interest.

## Publisher's Note

All claims expressed in this article are solely those of the authors and do not necessarily represent those of their affiliated organizations, or those of the publisher, the editors and the reviewers. Any product that may be evaluated in this article, or claim that may be made by its manufacturer, is not guaranteed or endorsed by the publisher.
